# Knowledge, attitudes, and practice of Malagasy physicians regarding atopic dermatitis

**DOI:** 10.1016/j.jdin.2023.09.002

**Published:** 2023-09-09

**Authors:** Fandresena A. Sendrasoa, Tantely Razafison, Lala S. Ramarozatovo, Fahafahantsoa Rapelanoro Rabenja

**Affiliations:** Department of Dermatology, Faculty of Medicine, University of Antananarivo, Antananarivo, Madagascar

**Keywords:** atopic dermatitis, KAP study, Madagascar, physicians

*To the Editor:* Atopic dermatitis (AD) is a chronic relapsing inflammatory dermatosis of multifactorial etiology, characterized by pruritus.^1^ Apart from therapeutic adherence, the success AD management depends also on the knowledge, attitude, and practice (KAP) of the physicians who manage it. So, this study aims to assess the KAP regarding AD in Malagasy doctors.

A cross-sectional study was conducted during 3 months (March to June 2021), including physicians (in the public or private sector) practicing in Antananarivo and Antsirabe. Data concerning KAP were collected by an anonymous questionnaire (a set of 16 questions). The KAP study made by Kouotou et al^2^ was used to establish scores of the questionnaire. Each KAP section has questions, each correct response is scored at 1, result is expressed as a percentage. The cut-off scores for the KAP assessment was arbitrarily determined. KAP are distributed as follows:

- Knowledge: poor (score <50%), moderate (score ≥50 and <85%), high (score ≥85%)
- Attitudes: wrong (score <50%), approximate (score ≥50 and <85%), right (score ≥85%)
- Practices: inadequate (score <50%), average (score ≥50 and <85%), adequate (score ≥85%).

Epi Info version 7.1.3 was used for the statistical analysis. χ^2^test was used to analyze qualitative variables, *P* < .05 is considered as statistically significant.

Among 130 physicians invited to participate in this study, 122 responded (response rate of 93.8%).

The mean age ± SD of respondents was 42 ± 12.56 years (min: 24; max: 76 years).

Demographic profile of the study participants and their general knowledge, practice are shown in Table I, Table II and II, respectively.

**Table I:** Demographic profile of the study participants

Demographics	*N* = 122 (%)
Gender	
Male	48 (39.3)
Female	74 (60.6)
Mean age ± SD, y	42 ± 12.5
City	
Antananarivo	114 (94)
Antsirabe	8 (6)
Years of experience	
<5 y	40 (32.7)
5-10 y	24 (19.7)
>10 y	58 (47.5)
Medical specialty	
General practitioner	64 (52.4)
Pediatrics	43 (35.2)
Dermatology	10 (8.2)
Allergy	5 (4.1)

**Table II:** General knowledge and practice of medical staff

Statements	*N* (%) correctly answered according to medical specialty			
	GP	Pediatrics	Dermatology	Allergy
Definition				
AD is an inflammatory disease	46 (71.8)	40 (93)	10 (100)	5 (100)
AD is chronic	44 (68.7)	41 (95.3)	10 (100)	5 (100)
Associated allergic disorders				
Asthma	47 (73.4)	40 (86.9)	10 (100)	5 (100)
Allergic rhinitis	51 (79.6)	40 (86.9)	10 (100)	5 (100)
Allergic conjunctivitis	44 (68.7)	38 (82.6)	10 (100)	5 (100)
Origin of AD				
Allergic	49 76.5)	41 (89.1)	8 (80)	4 (80)
Genetic	30 (46.8)	36 (78.2)	8 (80)	3 (60)
Main symptom				
Pruritus	62 (96.8)	43 (100)	10 (100)	5 (100)
Primary lesions				
Erythema	61 (95.3)	40 (86.9)	9 (90)	4 (80)
Xerosis cutis	37 (57.8)	35 (81.3)	10 (100)	4 (80)
Sites in children (<2 y)				
Face	59 (87.5)	43 (100)	10 (100)	5 (100)
Scalp	9 (14)	17 (39.5)	8 (80)	2 (40)
Upper and lower limbs	30 (46.8)	18 (41.8)	8 (80)	3 (60)
Sites in adults				
Trunk	28 (43.7)	15 (34.8)	7 (60)	3 (60)
Skinfolds	33 (51.5)	24 (52.1)	10 (100)	4 (80)
Information about prevention				
Dietary intervention	53 (82.8)	31 (72)	9 (90)	4 (80)
Avoidance of specific and unspecific provocation factors	50 (78.1)	35 (81.3)	10 (100)	4 (80)
Educational intervention	47 (73.4)	34 (79)	10 (100)	4 (80)
Clinical knowledge based on iconography of AD cases photos				
Photo 1	40 (62.5)	29 (67.4)	10 (100)	4 (80)
Photo 4	38 (59.3)	30 (69.7)	10 (100)	3 (60)
Photo 5	36 (56.2)	29 (67.4)	10 (100)	3 (60)
Photo 8	57 (89)	38 (88.3)	10 (100)	5 (100)
Photo 10	26 (40.6)	20 (46.5)	8 (80)	3 (60)
Photo 11	41 (64)	33 (76.7)	10 (100)	4 (80)
Photo 12	12 (18.7)	11 (25.5)	7 (70)	3 (60)
Number of AD cases seen per month				
0	19 (29.6)	11 (25.5)	0 (0)	0 (0)
1-10	45 (70.3)	32 (74.4)	5 (50)	1 (20)
11-20	0 (0)	0 (0)	1 (10)	4 (80)
>20 cases	0 (0)	0 (0)	4 (40)	0 (0)
Drug prescription for AD flares				
Topical corticosteroids	48 (75)	41 (95.3)	10 (100)	5 (100)
Emollient	49 (76.5)	30 (69.7)	5 (50)	2 (40)
Antihistamines	42 (64)	33 (76.7)	7 (70)	3 (60)
Administration modalities of topical corticosteroids				
Once daily	22 (34.3)	21 (48.8)	6 (60)	3 (60)
Twice daily	42 (65.6)	22 (51.1)	4 (40)	2 (40)
Duration of the topical corticosteroid for AD flares				
<2 wk	34 (53.1)	28 (65.1)	5 (50)	1 (20)
2 wk	9 (14)	6 (13.9)	5 (50)	3 (40)
1 mo	23 (35.9)	12 (27.9)	0 (0)	1 (20)
Drug prescription in AD with intense pruritus				
Antihistamines	28 (43.7)	30 (69.7)	10 (100)	4 (80)
Oral corticosteroids	36 (56.2)	16 (37.2)	0 (0)	1 (20)

*AD*, Atopic dermatitis*.*

Depending on the medical specialties, knowledge levels varied. A total of 34% of general practitioners and 28% of pediatric physician’s assistant had poor level of knowledge. Residents in dermatology, dermatologists, and allergists had higher level of knowledge and practice score compared with residents in pediatry, pediatricians, and general practitioners (*P* < .05). It may be attributed to their comprehensive dermatology training and the fact that dermatologists see more patients with AD than doctors in other specialties. This result supported that dermatology training was required for medical workers who handle eczema, as described by Le Roux et al.^3^ The KAP study conducted in Abha City (Saudi Arabia) revealed also that dermatology training course attendees have sufficient knowledge regarding management of common dermatological disorders compared with those who did not.^4^

This study found correlation between number of AD cases seen per month and KAP scores for AD (*P* < .05). KAP scores increase proportionally to the number of AD cases seen in consultation. Unlike potential experience related to the year of experience, the number of AD cases treated per month gives cumulative experience or the real experience. So, our results show in medical practice that cumulative experience is more significant than potential experience.^5^

Our study show the moderate knowledge level, the approximate attitude, and the average practice of Malagasy physicians regarding AD, indicating the need for additional effort for optimal management and more continued education.

## Conflicts of interest

None disclosed.
